# Discovery of the Genomic Region and Candidate Genes of the *Scarlet Red Flesh Color* (*Y^scr^*) Locus in Watermelon (*Citrullus Lanatus* L.)

**DOI:** 10.3389/fpls.2020.00116

**Published:** 2020-02-19

**Authors:** Na Li, Jianli Shang, Jiming Wang, Dan Zhou, Nannan Li, Shuangwu Ma

**Affiliations:** The Laboratory of Melon Crops, Zhengzhou Fruit Research Institute, Chinese Academy of Agricultural Sciences, Zhengzhou, China

**Keywords:** watermelon, flesh color, QTL, fine mapping, marker-assisted selection, candidate gene, carotenoids

## Abstract

The flesh color of watermelon (*Citrullus lanatus*) is an important fruit quality trait that helps to determine fruit attractiveness and is potentially beneficial to human health. Previous inheritance analyses determined that a single dominant gene, *Y^scr^*, produces the scarlet red flesh color rather than the coral red flesh color in watermelon. However, no genomic region or gene-based molecular markers for the locus *Y^scr^* have been reported thus far. In the present study, two high-density genetic maps and whole-genome variation detection aided by genome resequencing were first map the flesh color locus *Y^scr^* to a small region on chromosome 6 based on two independent populations derived from two scarlet red-fleshed lines and two coral red-fleshed lines. Two major quantitative trait loci located in the same genomic regions were identified in the F_2_ and BC_1_P_2_ populations and explained 90.36% and 75.1% of the phenotypic variation in flesh color, respectively. Based on the genetic variation in the two parental lines, newly developed PCR-based markers narrowed the *Y^scr^* region to 40 Kb. Of the five putative genes in this region, four encoded glycine-rich cell wall structural proteins, which implied that a new regulatory mechanism might occur between scarlet red- and coral red-fleshed in watermelon. Moreover, the genotypes of two newly developed InDel markers (InDel27_fc6 and InDel28_fc6) were completely consistent with the phenotypes in the F_2_ and BC_1_P_2_ populations and all 56 scarlet red-fleshed watermelon accessions. The results presented here provide valuable information for marker-assisted selection of flesh color breeding and the functional validation of candidate genes in watermelon.

## Introduction

Watermelon [*Citrullus lanatus* (Thunb.) Matsum. & Nakai] is enjoyed worldwide for its fleshy, sweet, and juicy fruit and is often consumed in hot weather. The flesh color of watermelon is an important trait for consumers, making the selection of fruit with brightly colored flesh a priority for watermelon breeders (Evans, 2008). Watermelon accessions exhibit a wide range of flesh colors, including red, canary yellow, salmon yellow, orange, and white. Moreover, red flesh has been reclassified into two distinct flesh colors, coral red and scarlet red (Gusmini and Wehner, 2006). The genetic basis of flesh color in watermelon is complex, and several loci are known to affect flesh color. Wehner summarized the flesh color genes (Wehner, 2012) *B* (yellow flesh) (Shimotsuma, 1963), *C* (canary yellow flesh) (Poole, 1944), *i-C* (inhibitor of canary yellow) (Henderson et al., 1998), *Wf* (white flesh) (Shimotsuma, 1963; Robinson et al., 1976), *Y^scr^* (scarlet red flesh) (Gusmini and Wehner, 2006), *Y^crl^* (coral red flesh) (Porter, 1937; Poole, 1944; Henderson, 1989; Henderson et al., 1998), *y°* (orange flesh), and *y* (salmon yellow flesh) (Henderson, 1989; Henderson et al., 1998).

In addition to inheritance studies, several quantitative trait loci (QTLs) mapping and gene cloning studies on flesh color have been published. An early study found two flesh color QTLs in group 2 and 8 in F_2_ and BC_1_ populations segregating red, canary yellow, and white flesh (Hashizume et al., 2003). With the release of the draft genome of watermelon (Guo et al., 2013) and the advent of next-generation sequencing, the development of comparative linkage maps and QTLs between different populations is possible. Two flesh color QTLs are located on chromosomes 2 and 4, and map-based cloning was performed based on the white-fleshed line and red-fleshed line (Zhang et al., 2014). *Cla005011* is considered a lycopene β-cyclase (*LCYB*) and candidate gene in the genomic region of chromosome 4. Another gene was narrowed down to a region of 1,200 Kb on chromosome 2. Several studies also identified major QTLs for lycopene content on chromosome 4 (Liu et al., 2015; Liu et al., 2016) and focused on gene *LCYB* (Bang et al., 2007; Bang et al., 2010). A major QTL for β-carotene accumulation located on 2.4 Mb of chromosome 1 was identified by a segregating population from a cross of orange-fleshed watermelon accession NY0016 and a yellow-fleshed line (Branham et al., 2017). QTL mapping for lycopene content was also applied in segregating populations from red (scarlet red)-fleshed and pink (coral red)-fleshed watermelon lines, but no stable QTL was identified (Fall et al., 2019). Most modern commercial watermelon cultivars have red flesh, but the genetic basis of red flesh is unclear. An inheritance study suggested that a single dominant gene, *Y^scr^*, produces the scarlet red flesh color rather than the coral red flesh color (Gusmini and Wehner, 2006). However, no genes for scarlet red or coral red flesh and no gene-based molecular markers have been reported for this trait.

Previously, we constructed a high density genetic map based on an F_2_ population from the coral red-fleshed line ZXG01478 and scarlet red-fleshed line 14CB11 (Shang et al., 2016). In the present study, two high-density genetic maps and whole-genome variation detection aided by genome resequencing were used to first map the flesh color locus *Y^scr^* to a small region on chromosome 6 based on two independent populations derived from two scarlet red-fleshed lines and two coral red-fleshed lines. Specifically, the main objectives of this study were as follows: (1) to perform the preliminary linkage mapping of the QTLs for flesh color in the F_2_ population; (2) to construst another high-density linkage map and perform QTL mapping for flesh color; (3) to fine map the major QTLs for flesh color using newly developed polymerase chain reaction (PCR)-based markers based on the high-coverage resequencing of two parental lines; (4) to analysis potential candidate genes; and (5) to validate the watermelon germplasm *via* two tightly linked InDel markers.

## Materials and Methods

### Plant Materials, Field Experiments, and Trait Evaluation

An F_2_ population of 93 individuals was developed from the F_1_ cross between ZXG01478 (a coral red-fleshed line) and 14CB11 (a scarlet red-fleshed line) (Shang et al., 2016). The corressponding recombinant inbred line (RIL) population of 106 lines (Li et al., 2018) was also constructed by single-seed descent for four generations to confirm the phenotype of F_2_ individuals. B47, a scarlet red-fleshed line, was crossed as the female parent to J, a coral red-fleshed line, and the resulting F_1_ plant backcrossed to J to create the BC_1_P_2_ population (J × (B47 × J)), which included 89 plants. A total of 87 accessions, 31 with coral red flesh and 56 with scarlet red flesh, were screened from National Mid-term Genebank for Watermelon and Melon in Zhengzhou Fruits Research Institute of the Chinese Academy of Agricultural Sciences (Zhengzhou, China). All watermelon accessions, F_2_ and RILs and backcross BC_1_P_2_ populations were planted at the Zhengzhou Fruits Research Institute of the Chinese Academy of Agricultural Sciences in Zhengzhou. Parents, F_1_ and watermelon accessions were grown in triplicate with 10 plants each. The F_2_, RIL and backcross BC_1_P_2_ populations were planted in a green house following essentially regular management practices throughout the growing season. In order to ensure the fruits were harvested at full maturity, we divided the individual or lines into early-maturing and late-maturing subgroups according to previous maturity data. The early-maturing and late-maturing fruits were harvested 35 and 40 days after manual pollination, respectively. Each fruit was cut lengthwise and immediately visually scored for flesh color based on parental lines with distinct coral red and scarlet red flesh. Pictures of fruits across the experiment were taken, allowing further confirmation of flesh color phenotypes during data analysis.

The goodness-of-fit tests for flesh color were performed based on χ^2^ testing of the expected segregation ratios using SAS.

### Restriction Site Associated DNA Sequencing (RAD-Seq) and Genotyping

The RAD protocol was employed as described by Baird et al. (2008). The enzymes and restriction fragment sizes were evaluated based on the reference genome sequence (ftp://cucurbitgenomics.org/pub/cucurbit/genome/watermelon/97103/v1/) (Guo et al., 2013). *MseI* was selected for RAD library construction. The library for Illumina sequencing was constructed from 200 ng of each DNA sample. All libraries were sequenced using Illumina HiSeq X Ten at Shanghai Major Biological Medicine Technology Co., Ltd.

For single nucleotide polymorphism (SNP) calling, the Burrows-Wheeler Aligner (Li and Durbin, 2009) was applied for sequence alignment between the individual reads and the reference genome sequence, Genome AnalysisToolKit (McKenna et al., 2010) was used to detect SNP loci, and SAMtools (Li et al., 2009) was used to filter out SNP loci. The filtering of SNP loci was based on three criteria: (i) an average sequence depth of < fivefold in the parents and < threefold in the progeny; (ii) no polymorphism between the parents; and (iii) heterozygosity in the parents.

### Linkage Map Construction

The poorly performing markers were removed before map construction, which missed an excess of more than 50% of the missing data in the BC_1_P_2_ population. Markers with significant segregation distortion (χ^2^ test, P < 0.05) were excluded from the subsequent linkage map construction. The construction of the linkage map was performed using JoinMap 5.0 (https://www.kyazma.nl/index.php/JoinMap) with a goodness-of-fit threshold of ≦ 5, a recombination frequency of < 0.4 and a minimum logarithm of odds (LOD) score of 2.0. All genetic distances were expressed in centimorgans (cM), as determined by the Kosambi function (Kosambi, 2016). Linkage groups were assigned to chromosomes based on published high-density genetic map for watermelon (Shang et al., 2016).

### Gene Mapping for Qualitative Traits

Earlier genetic mapping was performed using morphological traits (Lister and Dean, 1993), which implied that flesh color could be treated as a phenotypic marker in later gene mapping. In the present study, two different methods were performed. First, the high-density linkage map was reconstructed with SNP markers on the linkage map and the phenotype marker (Scr_P) using JoinMap 5.0 software (https://www.kyazma.nl/index.php/JoinMap) as described above. Second, the genome-wide QTL scanning was performed by adopting Bayesian model selection (Yi et al., 2007; Kosambi, 2016) in the package R\qtlbim (www.qtlbim.org) (Yandell et al., 2007), which analyses the QTL model for binary traits. The appropriate LOD threshold was determined by a permutation test of 1,000 repetitions (Churchill and Doerge, 1994). LOD scores corresponding to P = 0.01 were used to identify novel QTLs. The linkage maps and QTL presentation were drawn using MapChart software (Voorrips, 2002).

### Development of Insertion/Deletions (InDel) and SNP Markers

Genome-wide identification of InDels and SNPs between ZXG01478 and 14CB11 was performed by our laboratory (Li et al., 2018). The extraction of 500 base pairs (bp) before and after SNP/InDel loci was performed by a self-compiled script on Perl. For developing PCR-based dCAPS (derived cleaved amplified polymorphic sequences) markers, the web-based free software dCAP Finder 2.0 program (http://helix.wustl.edu/dcaps/dcaps.html) was used to find appropriate restriction enzymes for detecting SNPs (Neff et al., 2002). Primer 5.0 (Clarke and Gorley, 2001) and Oligo 7 (Rychlik, 2007) were used to design the appropriate PCR primer sets.

### Genotyping of PCR-Based Markers

DNA from all materials and populations used in the present study was extracted from young leaves using the Hi-DNAsecure Plant Kit (Tiangen Biotech, Beijing, China) according to the manufacturer’s instructions. PCR was performed in 25 μl reaction volumes containing 12.5 μl 2 × Power Taq PCR MasterMix (Bioteke, Beijing, China) with 10 μM primer (each) and approximately 50 ng of genomic DNA as a template. Thermocycling was initiated at 94°C for 5 min, followed by 35 cycles of 94°C for 20 s, 55°C for 1 min and 72°C for 30 s, with a final extension at 72°C for 5 min. The PCR products were separated on an 8% polyacrylamide gel and visualized by silver staining. Bioinformatics analysis.

Simple Modular Architecture Research Tool (http://smart.embl-heidelberg.de/) (Letunic et al., 2015; Letunic and Bork, 2018) was used to search known motifs in protein sequences. Multiple sequence comparison was performed using multiple sequence alignment (https://www.ebi.ac.uk/Tools/msa/muscle/) (Madeira et al., 2019). Genomics data was visualized with Integrative Genomics Viewer (Thorvaldsdottir et al., 2013).

### Quantitative Real-Time PCR (qRT-PCR) Analysis

Bathes of flesh tissue was cut into smaller pieces, immediately frozen in liquid nitrogen and stored at −80°C. For the qRT-PCR template, the reverse transcription reaction was performed using a PrimeScriptTM II 1st Strand cDNA Synthesis Kit (Takara, Beijing, China). qRT-PCR was performed using a Bio-Rad IQ5 with SYBR Green detection. Relative expression levels were evaluated using the 2-△△CT method. The watermelon actin gene was used as an internal control to normalize transcript levels. The primer details are shown in **Supplementary Table 1**. A cycling temperature of 57°C and the criterion of a single peak on the melting curve were used to confirm the specificity of designed primer pairs.

## Results

### Location of the *Y^scr^* Locus Using High-Quality Genetic Maps

An F_2_ population derived from ZXG01478 (a coral red-fleshed line) and 14CB11 (a scarlet red-fleshed line) was generated (Shang et al., 2016). All fruits in F_1_ generations had scarlet red flesh, suggesting that the scarlet red flesh color was dominant. Based on visual observation, the flesh color produced in the F_2_ populations was classified into two categories, scarlet red and coral red. Fruits in the F_2_ generation segregated 68:25 (scarlet red: coral red), which fit a 3:1 ratio (χ^2^ = 0.18, P value = 0.68). Another crossed population, BC_1_P_2_ from the scarlet red-fleshed line B47 and the coral red-fleshed line, J, was also evaluated to determine the flesh color inheritance. All fruits in the F_1_ generations had scarlet red flesh, and the BC_1_P_2_ population segregated 43:46 (scarlet red: coral red), showing that the data were consistent with a 1:1 expectation (χ^2^ = 0.10, P value = 0.75). These results suggested that scarlet red flesh in watermelon is controlled by a single dominant gene, which is in agreement with the previous genetic analysis of the dominance of the *Y^scr^* locus (Gusmini and Wehner, 2006).

**Figure 1:**
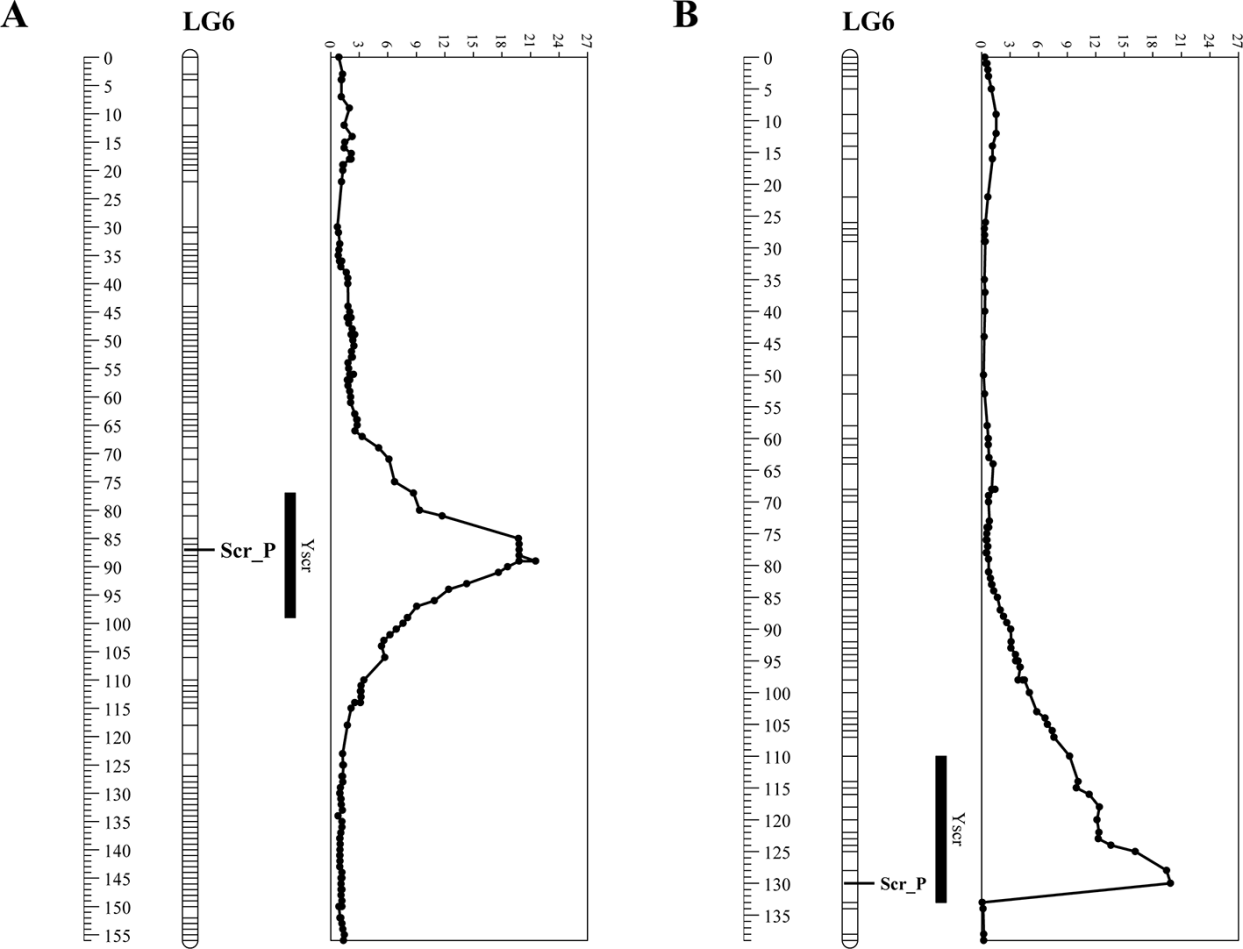
Linkage analysis of phenotypic marker for flesh color (Scr_P) and quantitative trait loci (QTL) profiling of *Y^scr^* on linkage groups in two populations. **(A)** Analysis based on the F_2_ population. **(B)** Analysis based on the BC_1_P_2_ population.

The degree of flesh color was used as a phenotypic marker (named Scr_P), the linkage map based on the F_2_ population, which covered a total of 2,634 SNP markers (Shang et al., 2016), was reconstructed. Scr_P was mapped to LG6 between Marker3917 and Marker3912 with genetic distances of 1 cM and 0.14 cM, respectively (**Figure 1A**). The genome-wide QTL analysis for flesh color was performed in the package R\qtlbim, which analyzes the QTL model for binary traits. For flesh color, one major QTL (*Y^scr^*), which explained 90.36% of the phenotype variation and showed a peak LOD of 23.65, was identified in linkage group 6 (**Figure 1A** and **Table 1**). The phenotypic marker, Scr_P, was located 1.95 cM from the genomic region with a peak LOD value. The SNP markers on the 1-LOD confidence interval showed significant association with flesh color (the average LOD = 14).

**Table 1:** Quantitative trait loci (QTL) mapping for flesh color in R\qtlbim, which analyzes the QTL model for binary traits.

Populations	QTL	Linkage groups	Peak position (cM)	LOD	R^2^	Flanking markers	Corresponding physical distance
F_2_	*Y^scr^*	LG6	88.68	23.65	90.36%	Marker3889-Marker3989	19.97–23.34 Mb
BC_1_P_2_	*Y^scr^*	LG6	139.92	20.36	75.10%	Mar6_19558234-Mar6_22960245	19.56–22.96 Mb

**Figure 2:**
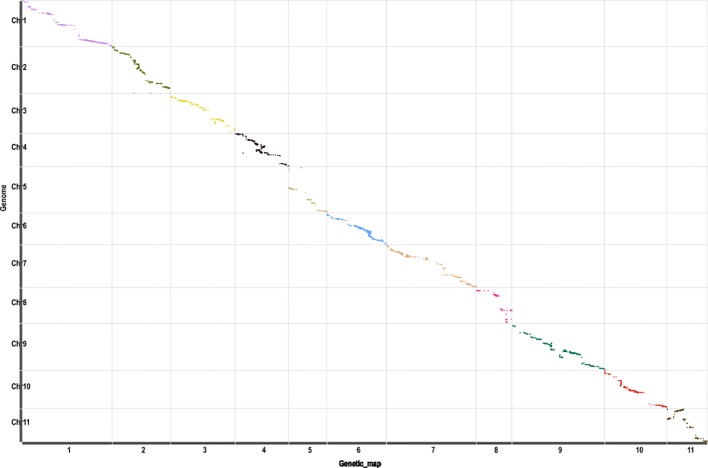
The collinearity of 11 linkage groups with the watermelon reference genome (97103 v1). The *x*-axis indicates the genetic distance of watermelon linkage groups accordingly, and the *y*-axis represents the linearity order of the physical position in the watermelon genome. All 5,808 SNP markers in these linkage groups are plotted as dots in the figure. Different colors indicate different linkage groups.

Another genetic map, which contained 5,808 SNP markers, was constructed (**Supplementary Table 2**) based on the RAD-seq data from B47, J and 89 BC_1_P_2_ individuals. The total length of the linkage map was 1,745.4 cM, with an average distance of 0.3 cM between adjacent markers. The relationship between the genetic map and physical maps was mostly linear for each linkage group except for part of LG11 (**Figure 2**), which was in agreement with the above linkage map. Scr_P was mapped to LG6 between Mar6_20935299 and Mar6_20935345 with genetic distances of 0.08 cM and 2.13 cM, respectively (**Figure 1B**). The genome-wide QTL analysis for flesh color was also performed in the package R\qtlbim. In the BC_1_P_2_ population, the major QTL (*Y^scr^*), which explained 75.1% of the phenotype variation and showed a peak LOD of 20.36, was identified (**Figure 1B** and **Table 1**).

### Fine Mapping of the *Y^scr^* Locus by PCR-Based Markers

To fine map the *Y^scr^* locus, InDels and SNPs in the QTL region were converted into PCR-based markers that could be rapidly and reliably analyzed. Genome-wide InDels and SNPs were investigated between ZXG01478 and 14CB11 based on the resequencing of the two parents (Li et al., 2018). A total of 106 InDels with a length greater than 8 bp were identified in the *Y^scr^* flanking regions (chromosome 6: 16.75-23.79 Mb). Of these, 24 markers for InDels with length ranging from 8 bp to 36 bp were developed (**Table 2** and **Supplementary Table 3**) to fine map the *Y^scr^* locus. Moreover, 1 dCAPS marker was developed based on SNPs located in the renarrowed region.

**Table 2:** The primer pair information for the PCR-based markers.

Marker name	F_primer (5′ to 3′)	R_primer (5′ to 3′)	Chr.	Position (bp)	InDel length	Restriction enzyme
InDel3_fc6	CGACTCATCGTTTCATCAAGA	GAACAGATTCGCTGGCAATAG	Chr6	20534344	35	
InDel5_fc6	GTTTCTCAAAATTGAGGCATT	AACTTGTATACTTGTTAGGCT	Chr6	20723762	25	
InDel6_fc6	AGGTATGTGACGCTCATTACG	TCTAAGTCGAGCGATCTTTTC	Chr6	20729791	22	
InDel7_fc6	CGACCATTTAAGCAAGCAGAT	GCAGTCATGAGCGTATGACTT	Chr6	20731970	20	
InDel8_fc6	CGATTCCTGTATAAAAGAATT	TACAATAATGGTGTTCAAGGT	Chr6	20740347	10	
InDel9_fc6	CAAAATCATTTCAATAAAGCAC	TGTTGTCTACCCTAAACCTAA	Chr6	21201052	50	
InDel10_fc6	ATAAGTTTGGTTATAGCGTTT	CGTATTTCTTTTATCACTTGG	Chr6	21213541	9	
InDel11_fc6	CCTCATTTCTCATAATAGTTT	CTCACCCTAATTTCTATTCCC	Chr6	21301055	12	
InDel13_fc6	TTTGCATAATAATTGAGGTGT	ATCCGCATAACTCGACATAAC	Chr6	21407047	12	
InDel14_fc6	CACAAATGCCAAACTTTCCTA	TTTCTTCTGCCAATGTATCTC	Chr6	21440621	25	
InDel16_fc6	GAAATAAAATTTGCTGTGACA	TTACATGAGCCCATACATAGA	Chr6	21625475	30	
InDel17_fc6	TACTTGTTCATTTGCCTCTAA	GGAGATATGCAAAGGGTCATG	Chr6	21639499	17	
InDel19_fc6	CTTTAATGCCTAATAACCAA	AAAGGAGGAAATTCAAATCA	Chr6	21671788	23	
InDel20_fc6	GGTTGAATCTGATTATTAGG	TTATAGCCAAGCTGCACATG	Chr6	21679346	8	
InDel21_fc6	TTTCTGTTTTGTCGGTTTCT	CACTCTACCAAATTAAAATCAA	Chr6	21741369	8	
InDel22_fc6	AATCTCACAAGTATGGAAGGT	CTTGGCTCAACTTACAAATAT	Chr6	21794530	15	
InDel23_fc6	ACAGAAGTGAAGCTTGGCAGA	ACGAGTTTGATGAAAATGGAA	Chr6	21818728	23	
InDel27_fc6	GACCCTGCATACGAGCCT	GGGATTGGAGCTGGAATT	Chr6	21861691	12	
InDel28_fc6	ATGGTAACCACCTATACCTTAA	ATACAATACAACCCAACCCAAT	Chr6	21862645	8	
InDel31_fc6	CGGTGAAACTCCATGAACAC	AAGCCTTTGAGACTCACAGGTC	Chr6	21891366	13	
InDel32_fc6	TTGAAGTTGGATTTGGTTGTT	AGTCCACCTTCTCATTTCCTG	Chr6	21909926	17	
InDel34_fc6	AAGATGTATCATAAATGTAAAGAC	GTTTGATAGATACTGATATTTTGT	Chr6	21918469	15	
InDel35_fc6	TTATCCTCCACTCTATTACTT	GAACTTTACTCTTTCATATTGCT	Chr6	21933128	15	
InDel37_fc6	GCCTTCATTGTAGCGTTCTGT	ATGGTCCATTTGGTGGGTTAA	Chr6	21945479	36	
dCAPS1_fc6	CCGTTTCCGGGCATATCCTC	GGTTCGCTTGATACGTCTGG	Chr6	21851079		TaqI

**Figure 3:**
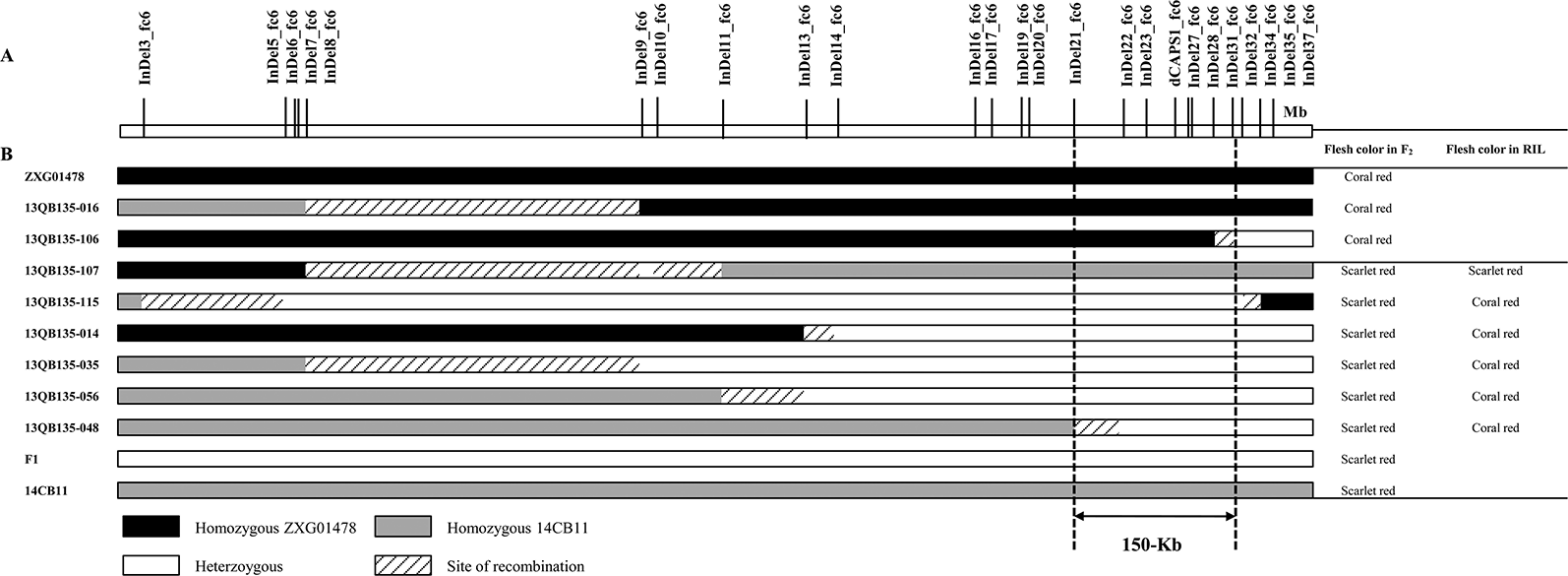
Graphical genotype of the recombinants in F_2_ and their flesh color in the F_2_ and recombinant inbred line (RIL) populations. **(A)** Partial physical map of the genomic region of *Y^scr^* on chromosome 6. **(B)** *Y^scr^* was narrowed to a 150 Kb region between InDel21_fc6 and InDel31_fc6 by analyzing the genotypes and phenotypes of the eight recombinants.

All 25 newly developed PCR-based markers were genotyped in the F_2_ population, and only eight recombinant individuals were identified around the *Y^scr^* narrowed region (**Figure 3**). Of the two recombinants with coral red flesh, 13QB135-016 showed a homozygous phenotype for coral red flesh downstream of InDel8_fc6 and delimited *Y^scr^* to a region downstream of InDel8_fc6; 13QB135-106 showed heterozygosity downstream of InDel31_fc6 and thus placed *Y^scr^* in a region upstream of InDel31_fc6. Of the six recombinants with scarlet red flesh in the F_2_ population, 13QB135-107 showed a homozygous phenotype for scarlet red flesh and the other five showed heterozygous phenotypes based on the phenotypes in the corresponding RILs. Therefore, 13QB135-107 showed heterozygosity upstream of InDel10_fc6 and placed *Y^scr^* in a region downstream of InDel10_fc6. As shown in **Figure 3**, 13QB135-115 placed *Y^scr^* in a region upstream of InDel35_fc6, 13QB135-014, 13QB135-035, 13QB135-056, and 13QB135-048 and delimited *Y^scr^* in a region downstream of InDel13_fc6, InDel8_fc6, InDel11_fc6, and InDel21_fc6. As a result, *Y^scr^* was narrowed down to a 150-Kb region between InDel21_fc6 (chromosome6: 21,741,369 bp) and InDel31_fc6 (chromosome6: 21,891,367 bp). Notably, allelic variation determined by five markers, InDel22_fc6, InDel23_fc6, dCAPS1_fc6, InDel27_fc6, and InDel28_fc6, cosegregated with Scr_P in the F_2_ population.

A total of 11 InDel markers and 1 dCAPs marker were genotyped in the BC_1_P_2_ population (**Supplementary Table 4**). Of the 43 fruits with scarlet red flesh, 17CB145-009 showed a parental homozygous genotype (scarlet red flesh) upstream of dCAPS1_fc6 and showed a maternal homozygous genotype (coral red flesh) downstream of InDel34_fc6, thus narrowing *Y^scr^* to a region between dCAPS1_fc6 and InDel34_fc6. Another 42 fruits showed a parental homozygous genotype (scarlet red flesh) upstream of dCAPS1_fc6 and delimited *Y^scr^* to a region downstream of dCAPS1_fc6. All 44 coral red-fleshed fruits in the BC_1_P_2_ population were heterozygous upstream of dCAPS1_fc6 and thus narrowed the *Y^scr^* locus to a region downstream of dCAPS1_fc6.

As a result of the two populations, *Y^scr^* was narrowed down to a 40-Kb region between dCAPS1_fc6 (chromosome6: 21,851,079 bp) and InDel31_fc6 (chromosome6: 21,891,367 bp) based on the 97103 reference genome (Guo et al., 2013).

### Candidate Genes for *Y^scr^*

**Figure 4:**
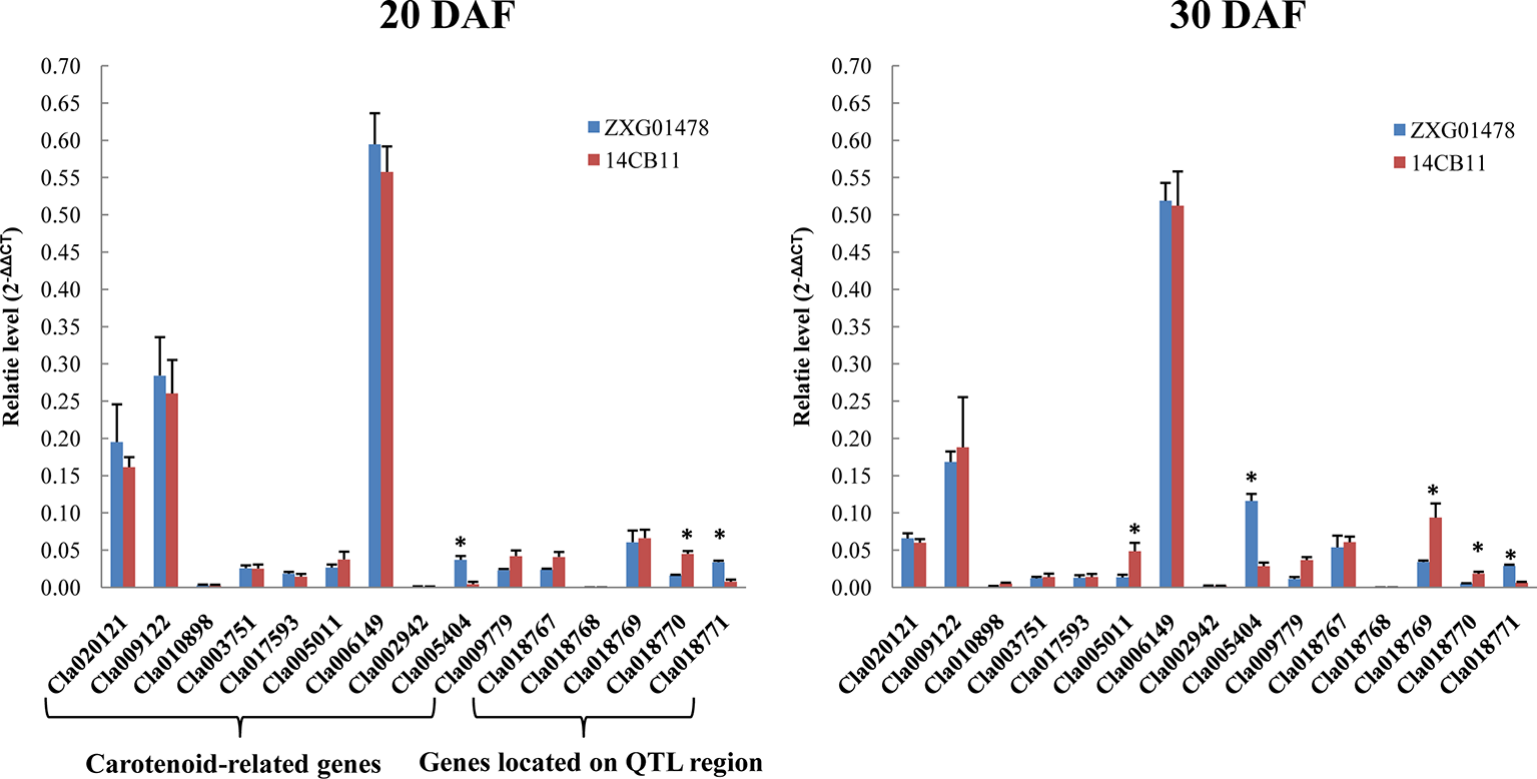
The expression profiles of carotenoid-related genes and potential candidate genes in flesh at different developmental stages using quantitative real-time polymerase chain reaction (qRT-PCR). DAF is the abbreviation for days after flowering. Watermelon actin was used as an internal control. Data are expressed as the means of the three biological replicates; error bars indicates SDs; ** indicates significance at p < 0.001.

According to the watermelon genome annotation (ftp://cucurbitgenomics.org/pub/cucurbit/genome/watermelon/97103/v1/), five putative genes (*Cla018767* to *Cla018771*) were annotated in the narrowed 40-Kb region (**Figure 4A**). Among the five genes, three (*Cla018768*, *Cla018769* and *Cla018771*) encoded unknown proteins. Both *Cla018767* and *Cla018770* encoded glycine-rich cell wall structural protein 2-like.

Protein sequences of the five putative genes revealed that *Cla018767 Cla018769*, *Cla018770*, and *Cla018771* had similar protein structures (**Supplementary Figure 1**). Simple Modular Architecture Research Tool identified a signal peptide, which was detected by the SignalP v4.0 program (Petersen et al., 2011), that started at position 1 and ended at positions 23, 23, 22, and 21 for the deduced proteins *Cla018767 Cla018769*, *Cla018770*, and *Cla018771*, respectively. Protein BLAST pairwise alignment against NCBI non-redundant (nr) protein sequences for these four signal peptides revealed significant hits with glycine-rich cell wall structural protein 2 (or -like). A region of low compositional complexity (residues 31–122, 35–133, 36–129, and 35–145 for the deduced proteins of *Cla018767*, *Cla018769*, *Cla018770*, and *Cla018771*, respectively), as detected by the SEG program (Wootton and Federhen, 1993), was also found, but no significant hits for this region were detected in the nr database.

### qRT-PCR for Candidate Genes and Carotenoids Related Genes

A total of 10 carotenoid-related genes, Cla020121 (GGPPS), Cla009122 (PSY), Cla010898 (PDS), Cla003751 (ZDS), Cla017593 (CRTISO), Cla005011 (LCYB), Cla006149 (CHYB), Cla002942 (NCED1), Cla005404 (NCED2), and Cla009779 (NCED3) were cloned in watermelon (Lv and Gu, 2013). Expression pattern of 10 carotenoid-related genes and five candidate genes located on the narrowed 40-Kb region in two parental lines, ZXG01478 (Coral red flesh) and 14CB11 (Scarlet red flesh), at differental developmental flesh tissues were investigated to analysis whether the expression level of these genes are associated with flesh color. As showed in **Figure 4**, 10 carotenoid-related genes were expressed in a similar pattern during watermelon fruit growth in two parental lines with coral red and scarlet red flesh, which was consistent with Lv and Gu’s study. Only three genes, Cla005404, Cla005011, and Cla018769 showed significantly different between two partnal lines for different stages tissuse. Notably, the expresison level of Cla018769, Cla018770, and Cla018771 showed significantly different among tissues, which might be the potential candidate genes.

### Validation of Watermelon Germplasm Using Two InDel Markers

**Figure 5:**
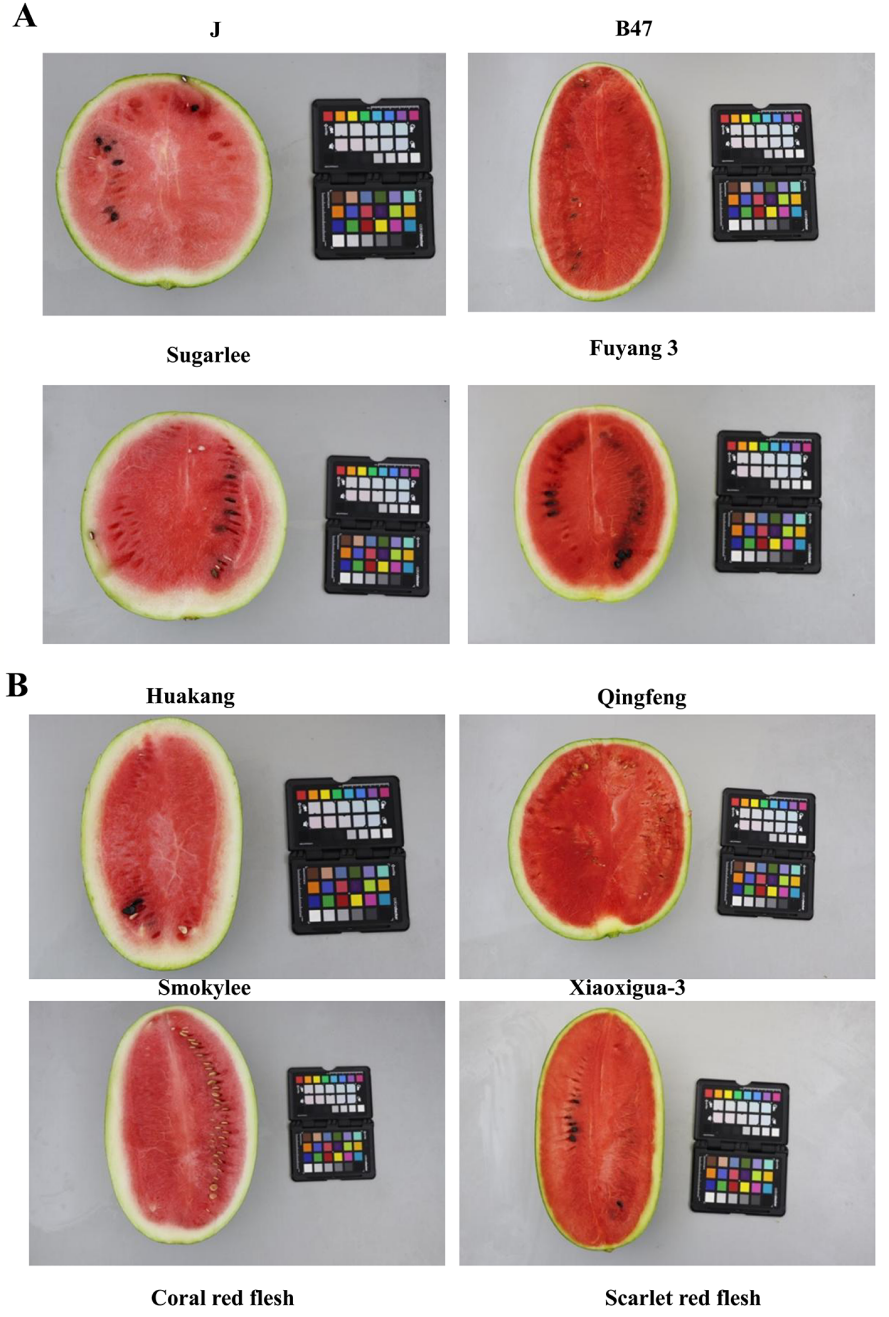
Photographs of longitudinal cross-sections of the parental lines and [the first line of **(A)**] a selection of the watermelon accessions with classic coral red- and scarlet red-fleshed color [the second line of **(A)** and **(B)**]. Left was the corel red-fleshed lines and right was the scarlet red-fleshed lines.

Two InDel markers, InDel27_fc6 and InDel28_fc6 cosegregated with flesh color in both the F_2_ and BC_1_P_2_ populations and thus were genotyped in a panel of 87 accessions with distinct coral red and scarlet red flesh (**Figures 5A, B**). As shown in **Table 3**, InDel27_fc6 and InDel28_fc6 cosegregated with the flesh color of all 87 accessions. Of the 31 accessions with coral red flesh, 24 showed maternal homozygous genotypes (coral red flesh, 167 bp and 197 bp for markers InDel27_fc6 and InDel28_fc6, respectively), and 7 showed parental homozygous genotypes (scarlet red flesh, 179 bp and 189 bp for markers InDel27_fc6 and InDel28_fc6, respectively). All 56 accessions with scarlet red flesh showed parental homozygous genotypes. In other words, all genotypes showed maternal homozygous genotypes (coral red flesh) with coral red flesh, while genotypes showed parental homozygous genotypes (scarlet red flesh), most with scarlet red flesh and several with coral red flesh. In summary, all genotypes matched the observed phenotype for accessions with coral red flesh, but not all genotypes matched the observed phenotypes for accessions with scarlet red flesh.

**Table 3:** Genotype identification of flesh color in 87 watermelon germplasm resources and four parental lines.

Accession name	Flesh color	InDel27_fc6	InDel28_fc6	Accession name	Flesh color	InDel27_fc6	InDel28_fc6	Accession name	Flesh color	InDel27_fc6	InDel28_fc6
ZXG01478^a^	Coral red	A (167 bp)	A (197 bp)	Jindu	Scarlet red	B	B	Yushan	Coral red	A	A
14CB11^b^	Scarlet red	B (179 bp)	B (189 bp)	Hongbaoshi	Scarlet red	B	B	Zhentouxigua	Coral red	A	A
J^c^	Coral red	A	A	Shenwu	Scarlet red	B	B	PI 270144	Coral red	A	A
B47^d^	Scarlet red	B	B	Zhengyin 65	Scarlet red	B	B	Calhoun Gray	Coral red	A	A
Meiguoxigua	Scarlet red	B	B	Feilvbintezao	Scarlet red	B	B	Fei88-110	Coral red	A	A
Xinfu	Scarlet red	B	B	Pingrang 1	Scarlet red	B	B	Foluolidajuren	Coral red	A	A
Fubao	Scarlet red	B	B	PI 612474	Scarlet red	B	B	PI 635712	Coral red	A	A
PI 635616	Scarlet red	B	B	Xibiqianxigua	Scarlet red	B	B	Meiguodahuapi	Coral red	A	A
PI 629104	Scarlet red	B	B	Yichui	Scarlet red	B	B	PI 479704	Coral red	A	A
Gaoyu	Scarlet red	B	B	Taiyangxigua	Scarlet red	B	B	Smokylee	Coral red	A	A
Huoxin	Scarlet red	B	B	Zhengyin 38	Scarlet red	B	B	Jubilee	Coral red	A	A
Miyako Nishiki	Scarlet red	B	B	Xiaoxigua-2	Scarlet red	B	B	PI 635594	Coral red	A	A
Kangwei	Scarlet red	B	B	Xin 1	Scarlet red	B	B	PI 635732	Coral red	A	A
Zaoban	Scarlet red	B	B	Zhengyin 43	Scarlet red	B	B	All Sweet	Coral red	A	A
Zhengyin 201	Scarlet red	B	B	Miandianxiaoxigua	Scarlet red	B	B	Long Crimson	Coral red	A	A
Qingfeng	Scarlet red	B	B	PI 635590	Scarlet red	B	B	Sangria	Coral red	A	A
Xiongyali 1	Scarlet red	B	B	Tedaxinhongbao	Scarlet red	B	B	Keniangriqi	Coral red	A	A
Xiongyin 2	Scarlet red	B	B	PI 306365	Scarlet red	B	B	Nongyoukangbing	Coral red	A	A
Luoning18rihong	Scarlet red	B	B	PI 537467	Scarlet red	B	B	Sugarlee	Coral red	A	A
Huadong 24	Scarlet red	B	B	Linyin 1	Scarlet red	B	B	Huakang	Coral red	A	A
Conglvhetaowen	Scarlet red	B	B	Mimei	Scarlet red	B	B	Fengguangfuben	Coral red	A	A
Fuyou	Scarlet red	B	B	French watermelon	Scarlet red	B	B	Zhongshengxi	Coral red	A	A
Wenlingxigua	Scarlet red	B	B	Guangxiheichang	Scarlet red	B	B	Paoteque Klondike	Coral red	A	A
Fuyang 3	Scarlet red	B	B	Zhengyin 30	Scarlet red	B	B	Hongliang 2	Coral red	A	A
Dujin	Scarlet red	B	B	Zhengyin 15	Scarlet red	B	B	Xiaozizaoxigua	Coral red	B	B
Red 1	Scarlet red	B	B	Zhengyin 64	Scarlet red	B	B	PI 502319	Coral red	B	B
Hongmei	Scarlet red	B	B	Spain	Scarlet red	B	B	PI 379247	Coral red	B	B
PI 612468	Scarlet red	B	B	Zhengyin 11	Scarlet red	B	B	PI 278013	Coral red	B	B
Wan87492	Scarlet red	B	B	Zhongbian 1	Scarlet red	B	B	PI 169242	Coral red	B	B
Wan87490	Scarlet red	B	B	Nongchong 1	Scarlet red	B	B	PI 319212	Coral red	B	B
								Anchanghong	Coral red	B	B

^a^The maternal parent of F_2_ population. ^b^The parental parent of F_2_ population. ^c^The maternal parent of BC_1_P_2_ population. ^d^The maternal parent of BC_1_P_2_ population.

## Discussion

Most modern watermelon cultivars have red flesh, and the intensity of the red color has become a preference for consumers when selecting fresh watermelon. The genetic base of different red flesh colors is unclear. In this study, using two high-density genetic maps and whole-genome variation detection aided by genome resequencing, the flesh color locus *Y^scr^* was first mapped on a region of ~ 40 Kb in chromosome 6 based on two independent populations derived from two scarlet red-fleshed lines and two coral red-fleshed lines. Moreover, the genotypes of two newly developed InDel markers (InDel27_fc6 and InDel28_fc6) were completely consistent with the phenotypes in the F_2_ and BC_1_P_2_ populations and all 56 scarlet red-fleshed watermelon accessions. The red carotenoid pigment lycopene accumulates in watermelon flesh and gives watermelon flesh its red flesh color (Di Mascio et al., 1989). However, no homologous carotenoid-related genes were found in the narrowed *Y^scr^* region. Moreover, carotenoid-related genes, including *GGPPS*, *PSY*, *PDS*, *ZDS*, *CRTISO*, *CCD*, *LCYB*, *CHYB*, and *NCED1*, were expressed in a similar pattern during watermelon fruit growth in red (scarlet red)- and pink (coral red)-fleshed watermelon in previous (Lv and Gu, 2013) and present studies. These results imply that a new regulatory mechanism might occur between scarlet red- and coral red-fleshed watermelon. Four putative genes encoded glycine-rich cell wall structural proteins on the narrowed *Y^scr^* genomic region and three (*Cla018769*, *Cla018770*, and *Cla018771*) showed significantly different expressed in different fruit stages between scarlet red- and coral red flesh lines. These genes contain a signal peptide followed by a region of low compositional complexity, including a high glycine-content region with (GX)_n_ repeats. Glycine-rich cell wall structural proteins as structural components of plant cell walls, appear to play important roles in signal transduction, protein-protein interactions, development, and transcriptional regulation, but there is no genetic evidencec of a specific function for these proteins (Ringli et al., 2001; Bocca et al., 2005). In addition, low-complexity sequences are extremely abundant in eukaryotic proteins (Marcotte et al., 1999) and may play a key role in the formation of novel genes (Toll-Riera et al., 2012). However, further evidence is needed to functionally validate the mechanism by which glycine-rich cell wall structural proteins control the flesh color in watermelon.

Great advances have been made in the analysis of carotenoids in watermelon. Ten cDNA fragments, geranylgeranyl pyrophosphate synthase (*GGPPS*, KF914758), *PSY* (KC166870), *PDS* (EF159942), *ZDS* (GQ140241), *CRTISO* (FJ788510), *LCYB* (EF014290), *CHYB* (FJ998045), and nine-cis-epoxycarotenoid dioxygenase (*NCED1*, FJ998046; *NCED2*, FJ998047; and *NCED3*, FJ998048), for carotenoid-related genes were cloned from watermelon (Lv and Gu, 2013). BLAST alignment against the reference genome coding sequences for these 10 carotenoid-related genes revealed significant hits with *Cla020121* (*GGPPS*), *Cla009122* (*PSY*), *Cla010898* (*PDS*), *Cla003751* (*ZDS*), *Cla017593* (*CRTISO*), *Cla005011* (*LCYB*), *Cla006149* (*CHYB*), *Cla002942* (*NCED1*), *Cla005404* (*NCED2*), and *Cla009779* (*NCED3*). Although *Cla003751* (chromosome6: 13,838,002–13,848,966 bp) and *Cla002942* (chromosme6: 9,157,470–9,159,170 bp) are located on chromosome 6, these two genes were not located within the *Y^scr^* narrowed genomic region and were located approximately 8 Mb and 12.8 Mb away from the QTL peak. Genetic analyses of flesh colors among red, orange, yellow, and white accessions have resulted in several advances and have indicated that flesh color might be associated with carotenoids in watermelon. A major locus for β-carotene accumulation is located on chromosome 1, and *Cla009122* is considered a phytoene synthase (*PSY*) and candidate gene (Branham et al., 2017). One gene for flesh color and lycopene content was mapped to the same region on chromosome 4 (Bang et al., 2007; Zhang et al., 2014; Liu et al., 2015), and *Cla005011* is regarded as the *LCYB* gene, which was identified as a key gene for color differentiation between red- and canary yellow-fleshed watermelon (Zhang et al., 2014).

Watermelon accessions exhibit a number of flesh color types, including colors that are easy to distinguish, such as red and white, and hard to distinguish, such as scarlet red and coral red. Limited researches on flesh color *via* molecular characterization and carotenoid profiles have focused on red, orange, yellow, and white (Bang et al., 2007; Zhang et al., 2014; Liu et al., 2015; Liu et al., 2016; Branham et al., 2017). Popular watermelon cultivars including Dixielee and Red-N-Sweet (Wehner, 2002) produce a more intense red color (scarlet red) than Angeleno Black Seeded, the type line for red flesh color (called coral red flesh to distinguish it from scarlet red). The first and only inheritance study between scarlet red and coral red flesh colors suggested that scarlet red flesh is controlled by a single dominant gene, *Y^scr^*(Gusmini and Wehner, 2006; Wehner, 2012). Because it is difficult to distinguish scarlet red and coral red colors when fruits are immature, scarlet red- and coral red-fleshed watermelons may have been miscategorized because previous categorizations didnot distinguish these two red colors. The National Mid-term Gene-bank for Watermelon and Melon was established in 2001 in our laboratory and has collected more than 3,000 watermelon and melon accessions from all over the world. After 15 years of collection, preservation, identification, and reproduction of these accessions, researchers in our laboratory had gained experience in fruit ripening and flesh color judgment. In addition to photo determination, coral red and scarlet red flesh could be distinguished by our researchers *via* standard materials with different red flesh. Therefore, the advantages of resources and phenotypic observations have enabled us to successfully identify genomic regions for flesh color that differentiate the coral red and scarlet red colors for the first time.

In summary, the present study first reported the genomic region *Y^scr^* associated with flesh color and identified potential candidate genes for this locus. Tightly linked markers were also developed and confirmed in different populations. The results presented here provide valuable information for marker-assisted selection of flesh color breeding and the functional validation of candidate genes in watermelon.

## Data Availability Statement

The data generated by this study can be found in Figshare (10.6084/m9.figshare.11647980).

## Author Contributions

NL and SM contributed to the conception and design of the study. NL, NNL, and JS performed the experiments. JS, SM, DZ, and JW contributed reagents/materials/tools and NL wrote the manuscript. All authors read and approved the final manuscript.

## Funding

This research was supported by the National Key R&D Program of China (2016YFD0100204-26), the National Natural Science Foundation of China (31601779 and 31872134), the Special Protection and Utilization of the Crop Germplasm Resources (2014NWB038), the National R&D Infrastructure and Facility Development Program of China (NICGR2015-016), and the Agricultural Science and Technology Innovation Program (CAAS-ASTIP-2018-ZFRI).

## Conflict of Interest

The authors declare that the research was conducted in the absence of any commercial or financial relationships that could be construed as a potential conflict of interest.

## Supplementary Material

Supplementary Figure 1 Clustal multiple sequence alignment result.

## Abbreviations

dCAPS, derived cleaved amplified polymorphic sequences; InDel, insertion/deletions; PCR, polymerase chain reaction; qRT-PCR, quantitative real time PCR; QTL, quantitative trait locus; RIL, recombinant inbred line; RAD-seq, restriction site associated DNA sequencing; SNP, single nucleotide polymorphism.
