# Supplementary figures and images for: Discovery of the Genomic Region and Candidate Genes of the Scarlet Red Flesh Color (Yscr) Locus in Watermelon (Citrullus Lanatus L.)
Source: Front Plant Sci. 2020 Feb 19;11:116. doi: 10.3389/fpls.2020.00116 (PMC7043143; doi:10.3389/fpls.2020.00116)

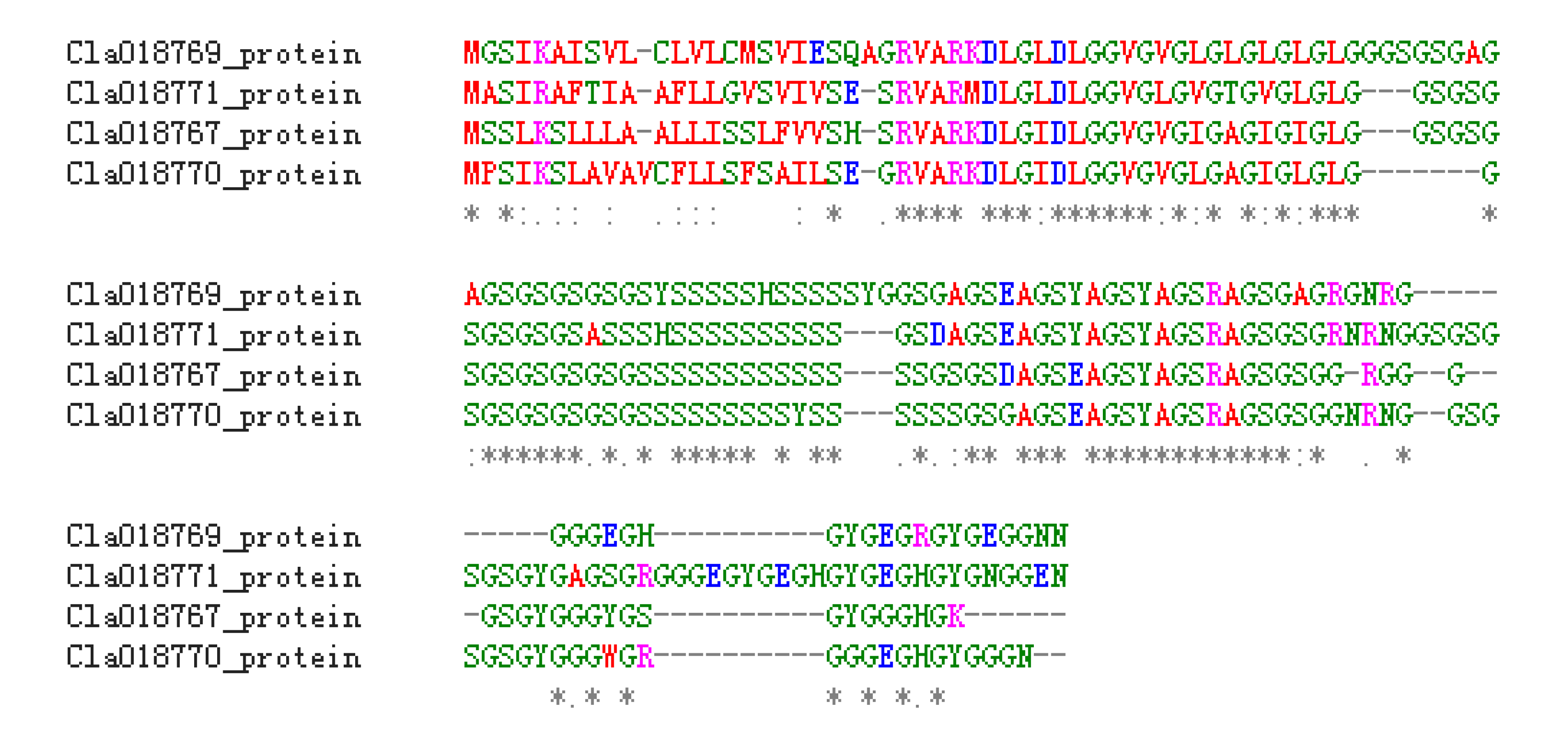

Supplement: Supplementary Figure 1 — Clustal multiple sequence alignment result. [file Image_1.tif]
